# Vick’s Floral Guide

**Published:** 1882-01-20

**Authors:** 


VICK'S FLORAL GUIDE and Catalogue of Choice Vegetable Seeds, etc., is on our table with its usual beautiful illustrations. His establishment is at Rochester, New York.

